# Construction and Identification of a Novel 5-Gene Signature for Predicting the Prognosis in Breast Cancer

**DOI:** 10.3389/fmed.2021.669931

**Published:** 2021-10-14

**Authors:** Lingling Guo, Yu Jing

**Affiliations:** ^1^Department of Ultrasound, The First Affiliated Hospital of Jinzhou Medical University, Jinzhou, China; ^2^Clinical Trial Ward of the First Affiliated Hospital of Jinzhou Medical University, Jinzhou, China

**Keywords:** gene signature, breast cancer, bioinformatics analysis, co-expression module, risk score

## Abstract

**Background:** Breast cancer is one of the most common malignancies in women worldwide. The purpose of this study was to identify the hub genes and construct prognostic signature that could predict the survival of patients with breast cancer (BC).

**Methods:** We identified differentially expressed genes between the responder group and non-responder group based on the GEO cohort. Drug-resistance hub genes were identified by weighted gene co-expression network analysis, and a multigene risk model was constructed by univariate and multivariate Cox regression analysis based on the TCGA cohort. Immune cell infiltration and mutation characteristics were analyzed.

**Results:** A 5-gene signature (GP6, MAK, DCTN2, TMEM156, and FKBP14) was constructed as a prognostic risk model. The 5-gene signature demonstrated favorable prediction performance in different cohorts, and it has been confirmed that the signature was an independent risk indicater. The nomogram comprising 5-gene signature showed better performance compared with other clinical features, Further, in the high-risk group, high M2 macrophage scores were related with bad prognosis, and the frequency of TP53 mutations was greater in the high-risk group than in the low-risk group. In the low-risk group, high CD8+ T cell scores were associated with a good prognosis, and the frequency of CDH1 mutations was greater in the low-risk group than that in the high-risk group. At the same time, patients in the low risk group have a good response to immunotherapy in terms of immunotherapy. The results of immunohistochemistry showed that MAK, GP6, and TEMEM156 were significantly highly expressed in tumor tissues, and DCTN2 was highly expressed in normal tissues.

**Conclusions:** Our study may find potential new targets against breast cancer, and provide new insight into the underlying mechanisms.

## Introduction

Breast cancer is one of the most frequently diagnosed malignancies in women and the major cause of cancer-associated mortality worldwide. Recently, the World Health Organization's International Agency for Research on Cancer released the latest global cancer burden data for 2020. The most obvious change is that the incidence of breast cancer has increased rapidly with 2.26 million new cases, thereby replacing lung cancer (2.20 million new cases) as the most common cancer (1). From 2012 to 2016, the incidence of breast cancer increased by 0.3% per year, and the mortality rate continued to decline (2, 3). Assessing and improving breast cancer patients' outcomes are still tasks of considerable importance.

In recent years, the diagnosis of BC mainly depended on pathological examination, imaging examination, and evaluation of tumor biomarkers. Because of the high recurrence rate of BC, the age of onset of BC become younger gradually (4). As a potential non-invasive monitoring option for the risk of recurrence in BC patients, gene signatures have attracted more and more attention. The integration of multiple biomarkers into a single model can improve the accuracy of prediction compared to a single clinical biomarker. Therefore, it is necessary and effective to construct new biomarkers related to the prediction of curative effect. The construction of such genetic markers may have the clinical potential to predict the prognosis of patients and aid in treatment selection. Previous studies have established prognostic signatures for breast cancer by bioinformatics, such as, Zhong et al. established an autophagy-related genes-based prognostic signature for breast cancer patients, which was of great significance in predicting the overall survival rate (5). Zhang et al. developed an 11-gene signature associated with glycolysis to predict survival in breast cancer patients (6). In addition, Xie et al. developed a 12-gene prognostic signature that provided new insights for assessing the high risk of death from breast cancer and individualized use of immunotherapy (7). Although several gene signatures associated with breast cancer have been published, some of them still have some defects, and the existing work related to prognosis of breast cancer patients has not been well carried out. Therefore, there is an urgent need to construct a breast cancer gene signature biomarkers to predict prognosis and optimize treatment.

This study aimed to identify prognostic differentially expressed genes (DEGs) and construct and validate a risk model for breast cancer. Moreover, the differences in immune cell infiltration and mutation character between high- and low-risk patients were evaluated. We built a 5-gene signature prognostic risk model with excellent stability and reliability for predicting prognosis in breast cancer patients.

## Materials and Methods

### Data Download and Preprocessing

The RNA-seq and clinical data of breast cancer were downloaded from The Cancer Genome Atlas (TCGA; https://portal.gdc.cancer.gov/). The RNA sequencing data were pre-processed in the following steps: (1) the samples without clinical data were removed; (2) the median expression value was selected for gene symbols corresponding to multiple probes.

The GSE59515 data set, which contained information on neoadjuvant ultrasound evaluating the sensitivity of drug resistance, and the GSE20685 and GSE31448 data sets, which contained information on the survival time of breast cancer, were obtained from the Gene Expression Omnibus (GEO). The GEO data sets were pre-processed in the following steps: (1) the samples without clinical data were removed; (2) the probes were converted to gene symbols; (3) the probes corresponding to more than 1 gene were eliminated; (4) the median expression value was selected for gene symbols corresponding to multiple probes. After preprocessing, there were a total of 50 samples, including 34 for drug response (responder) and 16 for non-response (non-responder), in the GSE59515 data set. There were 1,034 samples in the TCGA data set, 327 in the GSE20685 data set, and 246 in the GSE31448 data set. The clinical statistics information for all cohorts is shown in Table 1.

**Table 1:** Sample information.

**Clinical features**	**TCGA-BRCA**	**GSE20685**	**GSE31448**	**GSE59515**
**Response**				
Responder				34
Non-responder				16
**OS**				
0	888	244	167	
1	146	83	79	
**T stage**				
T1	176			
T2	591			
T3	128			
T4	36			
TX	3			
**N stage**				
N0	484			
N1	351			
N2	110			
N3	72			
NX	17			
**M stage**				
M0	851			
M1	21			
MX	162			
**Stage**				
I	179			
II	585			
III	229			
IV	19			
X	22			
**ER**				
Negative	222			
Positive	769			
Unknown	43			
**PR**				
Negative	317			
Positive	672			
Unknown	45			
**Her2**				
Negative	527			
Positive	151			
Unknown	356			
**Age**				
≤ 60	578			
>60	456			
**Subtype_PAM50**				
Basal	177			
Her2	73			
LumA	538			
LumB	196			
Normal	38			
Unknown	12			

### Identification of Differentially Expressed Genes Associated With Neoadjuvant Chemosensitivity and Functional Annotation

The limma package was applied to calculate the DEGs between the responder and non-responder subtypes in the GSE59515 data set. Further, the Gene Ontology (GO) functional enrichment and Kyoto Encyclopedia of Genes and Genomes (KEGG) pathway of the differentially expressed genes between the responder and non-responder groups were performed by the R package WebGestaltR (v0.4.3).

### Identification of Co-expression Modules

The weighted gene co-expression network analysis (WGCNA)was applied to identify co-expression genes and modules based on the GSE59515 expression profiles by the R software package. The log(k) of the node with connection degree k was inversely associated with the log(P(k)) of the probability of the occurrence of the node, and the genes with a correlation coefficient > 0.85 were included. Subsequently, the expression matrix was transformed converted into the adjacency matrix, and the topological overlap matrix was calculated from the adjacency matrix, and then hierarchical clustering was performed. Next, average linkage hierarchical clustering was performed on the basis of the topological overlap dissimilarity measure. After the gene module was determined, coexpressed modules were determined using a dynamic hybrid tree cut algorithm setting with a least number 100 for each module, and the eigenvectors of each module were calculated and the closer modules were merged into a new module.

### Identification of Hub Genes and Protein-Protein Interaction Network Analysis

STRING (https://string-db.org/) is a public database that contains interactions between known and predicted proteins, covering 9.6 million proteins and 13.8 million protein-protein interactions from more than 2,031 species. STRING is a comprehensive database derived from experimental data, co-expression data, and automated text mining, and it also contains the results of bioinformatics predictions. The study of the protein-protein interaction network is helpful to excavate hub regulatory genes. There are many databases of protein-protein interactions, but STRING covers the most species and has the most information about interactions.

We identified the common DEGs between the responder, non-responder, and yellow module genes, and then we drew a Venn diagram. We analyzed the protein-protein interaction (PPI) network of these common genes by the STRING database and performed visualization with Cytoscape (v3.7.2) to find the network module.

### The Prognostic Risk Model Construction

#### Training Set Samples Random Grouping

First, all samples (*n* = 1,034) from the TCGA database were randomly assigned to a training cohort and a validation cohort at the ratio of 1:1. To eliminate the influence of random allocation bias on the model stability, samples were randomized by resampling 100 times with replacement. Eligible training and validation cohort were selected according to the following criteria: (1) both groups were well-matched for age, sex, follow-up time, and patient mortality ratio; and (2) the numbers of binary classification samples were close after clustering the gene expression profiles into 2 randomly grouped data sets.

#### Univariate Survival Analysis of the Training Cohort

Univariate survival analysis was assessed using the R survival coxph function (*P* < 0.05) to identify the prognostic hub genes in the training cohort. Stepwise regression based on Akaike Information Criterion (AIC) was used to provide a balance between the goodness of model fit and the number of parameters required. Variables were dropped sequentially by evaluating the effects of their removal on the models AIC, where the lower the AIC, the better the fit.

### Immunohistochemical Staining Evaluation

To validate the expression of 5 gene ssiganture, tissue microarrays comprised of 89 cases (45 cases of BRCA tissues, 44 cases of normal paired samples) were purchased from Shanghai Outdo Biotech Co., Ltd. The studies were conducted in accordance with the International Ethical Guidelines for Biomedical Research Involving Human Subjects (CIOMS), and the research protocols were approved by the Clinical Research Ethics Committee of First Affiliated Hospital of Jinzhou Medical University.

The TMA slides were incubated with anti-GP6 antibody (1:200 dilution; SAB - 47582), FKBP14 antibody (1:100 dilution; GENX SPAN- GXP155296), DCTN2 antibody (1:100 dilution; GENX SPAN- GXP187676), MAK antibody (1:100 dilution; GENX SPAN- GXP309295), TMEM156 antibody (1:100 dilution; Proteintech-25159-1-AP), and spend the night at 4C.

The stained score were evaluated by three pathologists who were blinded to patients' clinical characteristics. The scoring system was based on the proportion of positive cells in all tissue cells and the staining intensity of these positive cells. The intensity of staining was classified as 0 (negative), 1 (weak), 2 (moderate), or 3 (strong). The staining ratio of positive cells was: 0 (<5%), 1 (5–25%), 2 (26–50%), 3 (51–75%), or 4 (> 75%). According to the staining intensity and the proportion of positive cells, the immunohistochemical results were divided into 0–1 grade, negative (–); > 1–4, weakly positive (+); > 4–8, moderately positive (++), and > 8–12, strong positive (+++).

## Results

### Identification of Differentially Expressed Genes and Functional Enrichment

**Figure 1:**
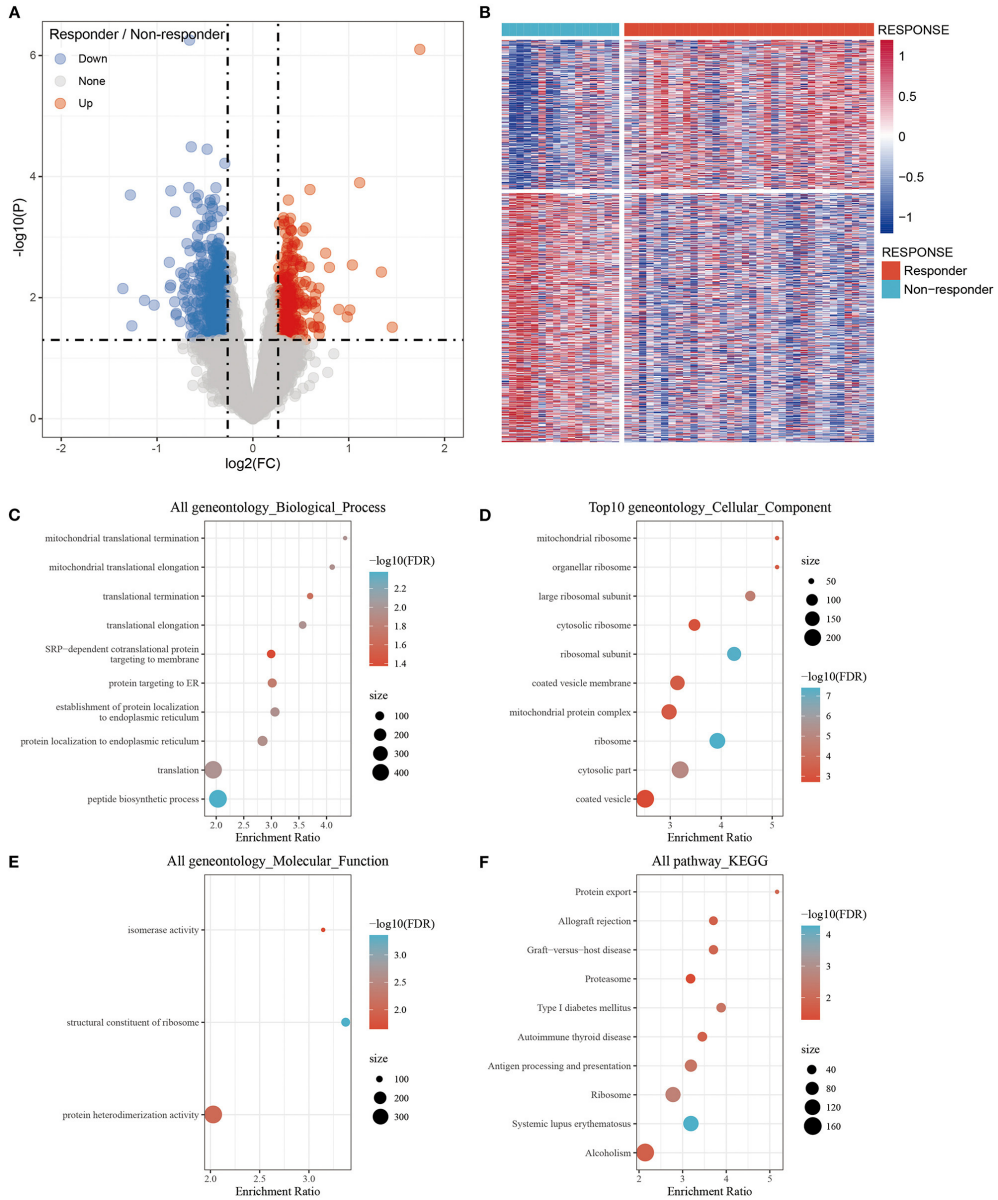
Identification of differentially expressed genes between the Responder and Non-responder group and functional enrichment. **(A)** Volcano plot, red represents up-regulation, and green represents down-regulation. The abscissa represents log2FC, and the ordinate represents *p*-value; **(B)** Heat map of differentially expressed gene expression of Responder and Non-responder group; **(C)** Differentially expressed genes enriched in Biological process; **(D)** Differentially expressed genes enriched in Cellular component; **(E)** Differentially expressed genes enriched in Molecular function; **(F)** Differentially expressed genes enriched in pathways. The abscissa represents the percentage of gene enrichment; the ordinate represents the enriched function or pathway.

The DEGs were filtered with thresholds of *P* < 0.05 and Fold Change (FC) >1.2. There were 979 DEGs including 367 upregulated and 612 downregulated genes between the responder and non-responder groups were identified. The volcano plot and heat map showed that the DEGs between the responder and non-responder groups were mainly downregulated genes (Figures 1A,B). The DEGs were listed in Supplementary Table 1.

The 979 DEGs between the responder and non-responder groups were subjected to GO functional enrichment analysis and KEGG pathway analysis by the R package WebGestaltR (v0.4.3). There were 10 significantly enriched biological process annotation terms with a False Discovery Rate (FDR) smaller than 0.05 presented (Figure 1C); 56 significantly enriched cellular component annotation terms (Figure 1D); and 3 significantly enriched molecular function annotation terms (Figure 1E). Besides, 10 KEGG pathways were identified (Figure 1F, Supplementary Table 2).

### Identification of Co-expression Modules

**Figure 2:**
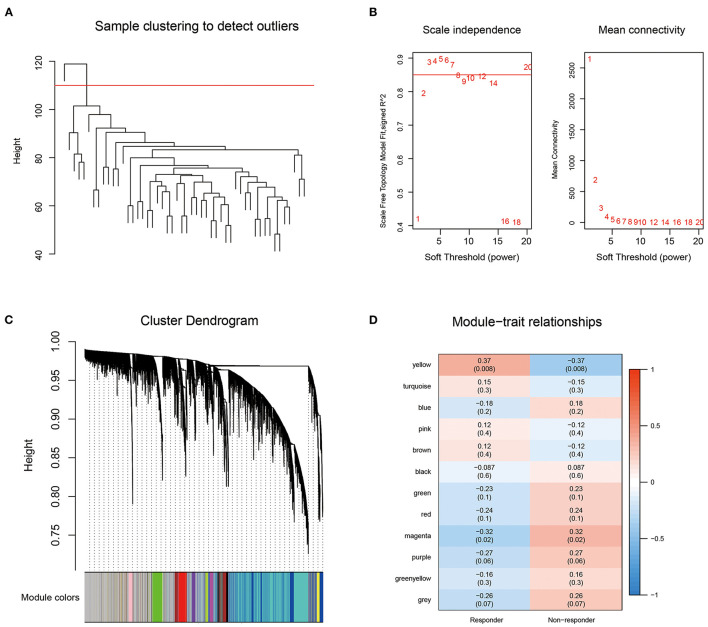
**(A)** Sample clustering analysis; **(B)** Network topology Analysis for various soft-thresholding powers; **(C)** The gene dendrogram and the corresponding module colors; **(D)** The correlation analysis between 12 modules clinical phenotypes.

First, we obtained the 1,034 breast cancer samples from the TCGA database and used hierarchical clustering to cluster the samples (Figure 2A). There was 1 outlier sample, which we removed, so 49 samples remained. Next, we calculated the corresponding Pearson correlation coefficients for each gene and built a weighted co-expression network using R software package WGCNA. Here, the power of β = 3 was selected ensuring close to the scale-free network (Figure 2B). After the module was determined by the dynamic tree cut algorithm, the eigenvectors of each module were calculated. Then we clustered the modules, merged the similar modules into new modules, and set height = 0.25, deepSplit = 3, minModuleSize = 100. Finally, we obtained 12 modules (Figure 2C). Further analysis of the correlations between each module and response type (responder and non-responder) was performed (Figure 2D). The results showed that the yellow module, which contained 697 genes (Supplementary Table 3), was the most positively correlated with the responder group and the most negatively correlated with the non-responder group.

### Identification of Hub Genes and Protein-Protein Interaction Network Analysis

We plotted the Venn diagram of the DEGs between the responder and non-responder groups and the yellow module genes, and 180 genes were identified as common genes, among which 112 were upregulated and 68 were downregulated in the responder group (Supplementary Figure 1A). Functional annotation was performed for these 180 genes (Supplementary Table 4).

The protein-protein interactions of the 180 DEGs were analyzed using STRING and the resulting file was used to screen the network modules using Cytoscape (v3.7.2). Then, the MCODE1 gene module was identified via the Molecular Complex Detection (MCODE) plugin (Supplementary Figure 1B). We performed functional annotation for the MCODE1 genes shown in Supplementary Table 5.

### The Prognostic Risk Model Construction

#### Training Set Samples Random Grouping

There were 517 samples in the training cohort and 517 in the validation cohort (Table 2). The result showed that the groupings were reasonable and all comparisons between the training and validation cohort were not significantly different by chi-square test (*P* > 0.05).

**Table 2:** TCGA training cohort and validation cohort sample information.

**Clinical Features**	**TCGA-train**	**TCGA-test**	***P***
**OS**			
0	446	442	0.7888
1	71	75	
**T stage**			
T1	145	31	0.5596
T2	291	300	
T3	65	63	
T4	14	22	
TX	2	1	
**N stage**			
N0	232	252	0.5106
N1	189	162	
N2	52	58	
N3	36	36	
NX	8	9	
**M stage**			
M0	428	423	0.861
M1	11	10	
MX	78	84	
**Stage**			
I	91	88	0.8007
II	297	288	
III	107	122	
IV	11	8	
X	11	11	
**ER**			
Negative	105	117	0.6178
Positive	389	380	
Unknown	23	20	
**PR**			
Negative	157	160	0.8921
Positive	336	336	
Unknown	24	21	
**Her2**			
Negative	263	264	0.9902
Positive	75	76	
Unknown	179	177	
**Age**			
≤ 60	292	286	0.7541
>60	225	231	
**Subtype_PAM50**			
Basal	89	88	0.9582
Her2	37	36	
LumA	264	274	
LumB	99	97	
Normal	21	17	
Unknown	7	5	

#### Univariate Cox Analysis of the Training Cohort

The R survival coxph function was applied to the 180 hub genes. There were 8 genes with a significant difference with *P* < 0.05 (Supplementary Table 6).

#### Multivariate Analysis of the Training Cohort

Eight prognosis-related genes were identified and then subjected to multivariate analysis and the best model with the lowest AIC was identified. Finally, 5 genes, *GP6, MAK, DCTN2, TMEM156*, and *FKBP14* were obtained.

The 5 genes prognostic Kaplan-Meier curves were shown in Supplementary Figure 2. The GP6, DCTN2, and TMEM156 genes could significantly divide the TCGA training cohort into high-risk and low-risk groups (*P* < 0.05). The final risk score formula was generated based on the hub genes as follows: risk score = −0.462^*^GP6-0.403^*^MAK+0.569^*^DCTN2-0.214^*^TMEM156+0.417^*^FKBP14.

#### Construction and Evaluation of the Risk Model

**Figure 3:**
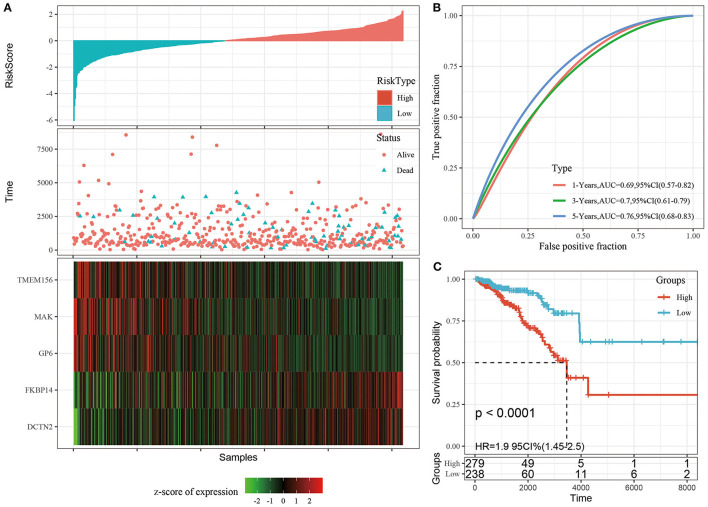
**(A)** The distribution of risk score and survival time, survival status as well as the 5 genes expression in the TCGA training cohort; **(B)** ROC curves and AUCs of the 5-gene signature classification performance in the TCGA training cohort; **(C)** KM curve of 5-gene signature in the TCGA training cohort. The abscissa represents survival time, the ordinate represents survival probability, the red line represents high expression group, and the green line represents low expression group.

We calculated and visualized the each sample risk score (Figure 3A). It was considered that breast cancer patients in the high-risk group had a worse prognosis. The 5 signature gene expression values changed with increased risk values. Further, the receiver operating characteristic curves (ROC) of risk score was plotted with the R package timeROC. The areas under the curve (AUCs) at 1, 3, and 5 years were 0.69, 0.70, and 0.76, respectively (Figure 3B). Patients were divided into the high-risk group (risk scores >0) and low-risk group (risk scores <0) using the Z-score method. Significant differences were found in the high-risk group with 297 samples and the low-risk group with 238 samples by KM curves (*P* < 0.0001, Figure 3C).

### Validation of the Risk Model

#### Validation of the Robustness of the 5-Gene Signature in Different Cohorts

To determine the model's robustness, we used the same coefficients and formul as the training set in the entire TCGA cohort. We calculated the risk score of each sample according to the gene expression level, and draw their distribution in the testing cohort as shown in Supplementary Figure 3A. The risk score at 1, 3, and 5 years with ROC was plotted (Supplementary Figure 3B). Significant differences were found in the high-risk group and the low-risk group by KM curves, and patients in the high-risk group had a significantly worse prognosis (*P* < 0.01, Supplementary Figure 3C).

The external cohorts GSE20685, GSE6532, and GSE42568 were included to test the model robustness. We calculated and plotted the risk score of each sample in the GSE20685, GSE6532, and GSE42568 cohorts (Supplementary Figures 3D,G,J). ROC of Risk score was plotted with the R package timeROC. The 3-, and 5-year ROC results of the GSE20685 was 0.70,0.66, respectively (Supplementary Figure 3E). The 3-, and 5-year ROC results of the GSE6532 was 0.78, 0.70, respectively (Supplementary Figure 3H). And the 3-, and 5-year ROC results of the GSE42568 was 0.66, 0.74, respectively (Supplementary Figure 3K). Significant differences were found in the KM curves between the high-risk and low-risk groups in the GSE20685, GSE6532m and GSE42568 cohorts (*P* < 0.05, Supplementary Figures 3F,I,L).

### Prognostic Analysis of the Risk Model and Clinical Features

**Figure 4:**
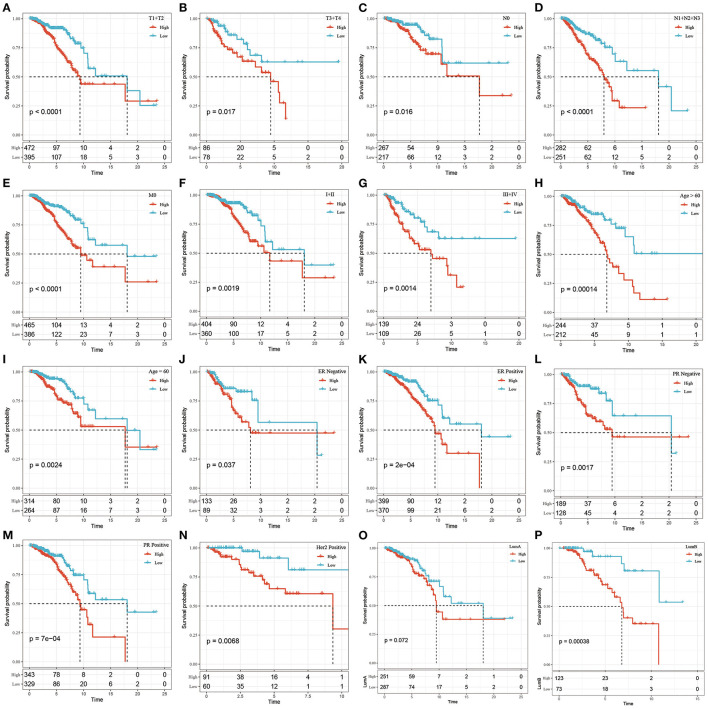
The prognostic performance of risk signature on different clinical features in the TCGA dataset. **(A)** T1+T2 group of patients; **(B)** T3+T4 group of patients; **(C)** N0 group of patients; **(D)** N1+N2+N3 group of patients; **(E)** M0 group of patients; **(F)** Stage I+II group of patients; **(G)** Stage III+IV group patients; **(H)** Age> 60 group patients; **(I)** Age ≤ 60 group patients; **(J)** ER negative group patients; **(K)** ER postive group patients; **(L)** PR negative group patients; **(M)** PR postive group patients; Patients in **(N)** Her2 postive group; patients in **(O)** LumA group; patients in **(P)** LumB group. The abscissa represents survival time, the ordinate represents survival probability, the red line represents high expression, and the green line represents low expression.

We found that the tumor stage (T stage), node stage (N stage), metastasis stage (M stage), clinical stage, ER status, PR status, HER2 status, and the LumB subtype could be divided into 2 groups with significant prognostic differences based on the risk score (*P* < 0.05, Figure 4). However, the risk score could not significantly divide the LumA subtype into 2 groups (*P* > 0.05, Figure 4O). These results demonstrated that Riskscore can be used as a prognostic marker for clinical subgroups.

### Performance of the Risk Score in Clinical Features

**Figure 5:**
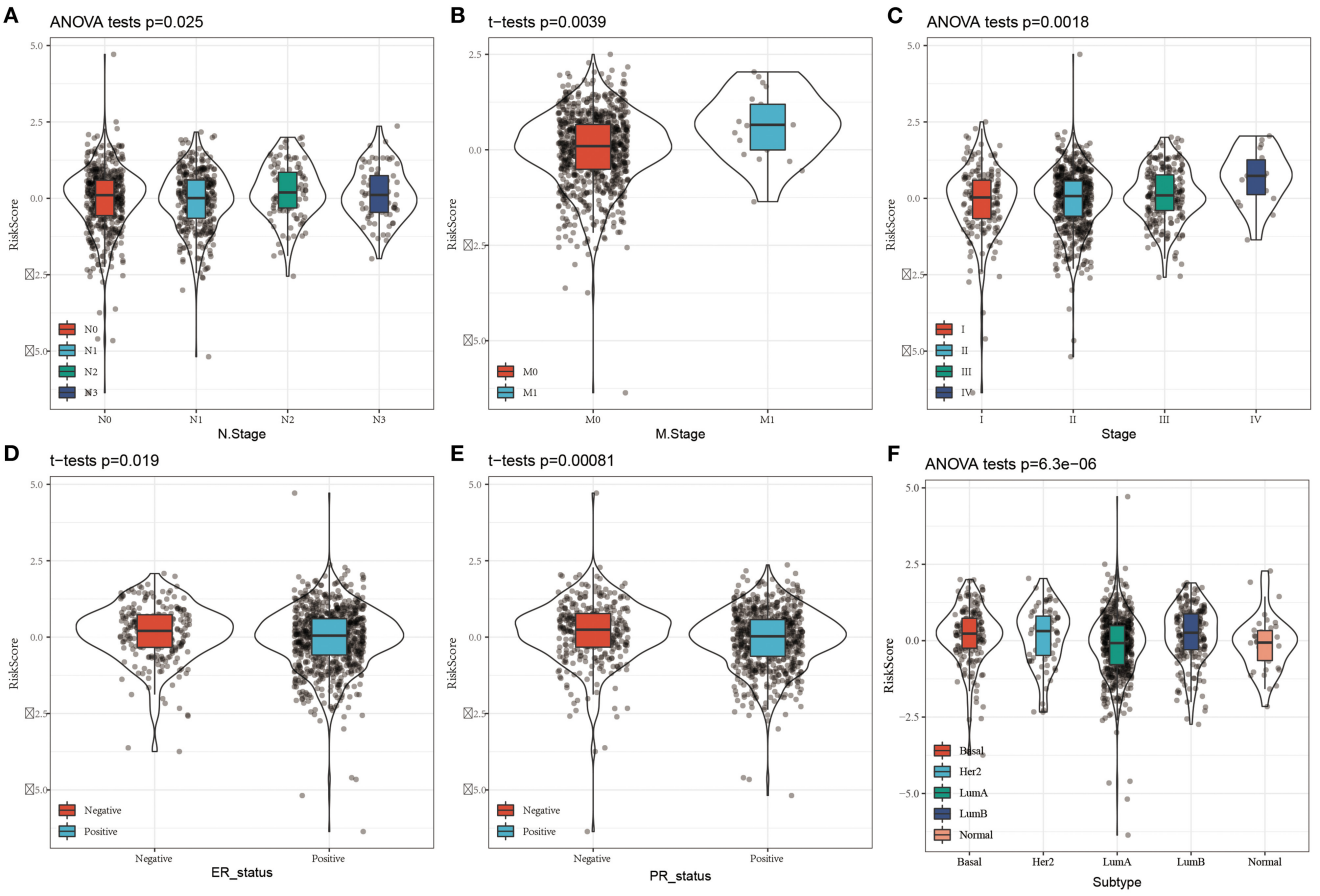
Distribution of Risk score on different clinical features in the TCGA dataset. **(A)** The expression difference of Risk score between N stages; **(B)** The expression difference of Risk score between M stages; **(C)** The expression difference of Risk score between clinical stages; **(D)** The expression difference of Risk score between ER status; **(E)** The expression difference of Risk score between PR status; The expression difference of FD Risk score between different molecular subtypes.

By comparing the distributions of the risk scores among the clinical features in the TCGA cohort, we found that there were significant differences in N stage, M stage, stage, ER status, PR status, and the PAM50 subtype (*P* < 0.05, Figure 5).

### Comparison of Immune Scores Between the High and Low-Risk Groups

**Figure 6:**
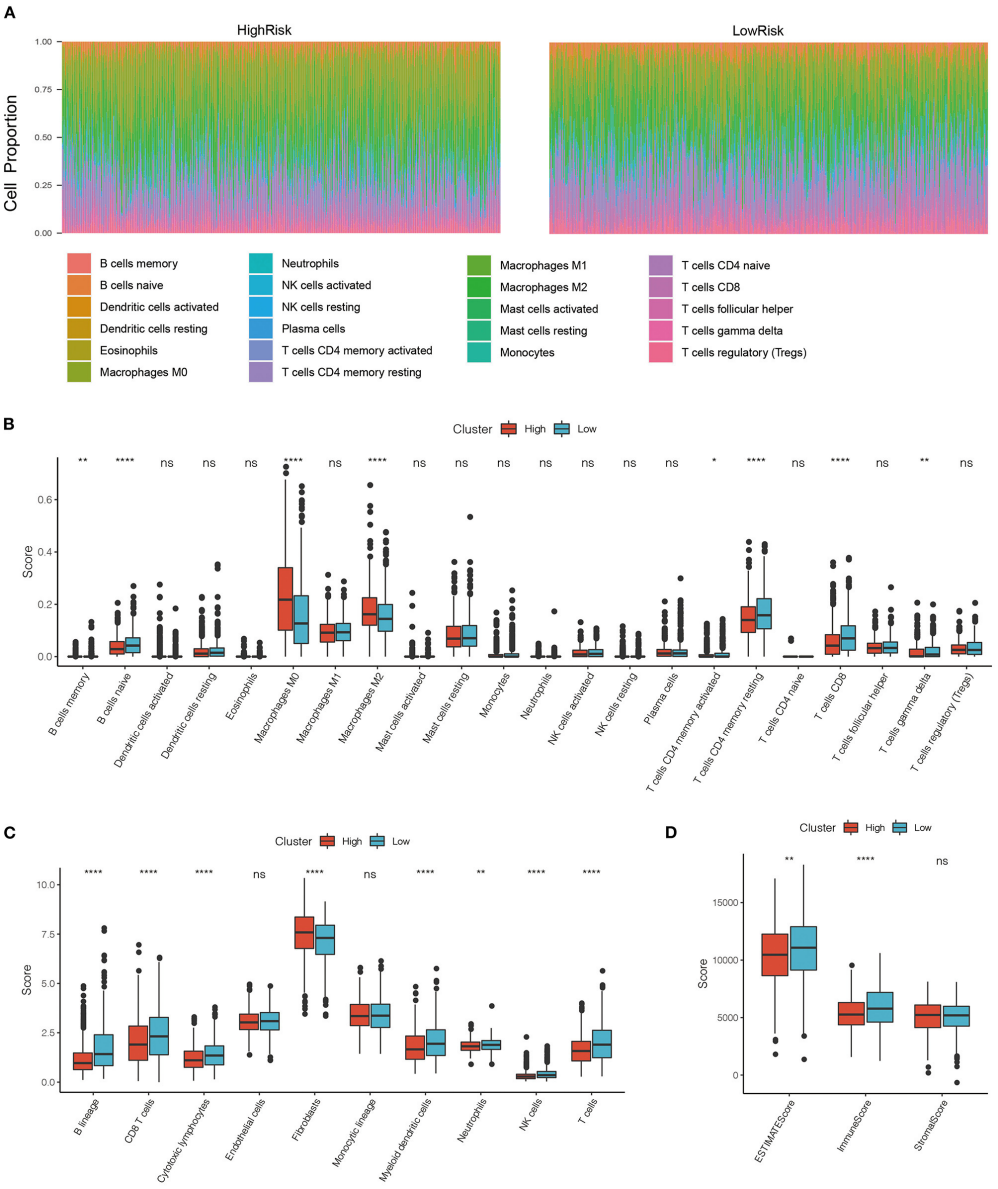
**(A)** Distribution of immune cell infiltration score in high and low-risk groups. **(B)** Comparison of immune scores between high risk and low-risk group in CIBERSORT; **(C)** Comparison of immune scores between high risk and low-risk group in MCPcounter; **(D)** Comparison of immune scores between high risk and low-risk group in the estimate. *P < 0.05, **P < 0.01, and ****P <0.001.

To determine the relationship between the immune scores and high-risk and low-risk groups of the TCGA cohort, we evaluated stromal scores, immune scores, and estimate scores by the ESTIMATE package, assessed the scores of 10 immune cell types by MCPcounter, and calculated the proportion 22 immune cell types by CIBERSORT. We then compared the differences between immune scores and the risk score groups. The distributions of the high- and low-risk scores in terms of the 22 immune infiltration cell scores were shown in Figure 6A. Box plots showed that M0 macrophages and M2 macrophages had significantly higher scores in the high-risk group than in the low-risk group, while B cells and CD8+ T cells had significantly lower scores in the high-risk group than in the low-risk group (Figure 6B). The scores of B cell and T cells CD8 in the high-risk group were significantly lower than in the low-risk group, which was consistent with the CIBERSORT. In addition, the scores of myeloid dendritic cells, neutrophils, and cytotoxic lymphocytes were significantly lower in the high-risk group than in the low-risk group (Figure 6C), and the immune scores and estimate scores in the low-risk group were higher than in the high-risk group (Figure 6D). To a certain extent, different immune expression characteristics affected the prognosis of different groups.

### Comparison of Mutation Characteristics Between the High- and Low-Risk Groups

**Figure 7:**
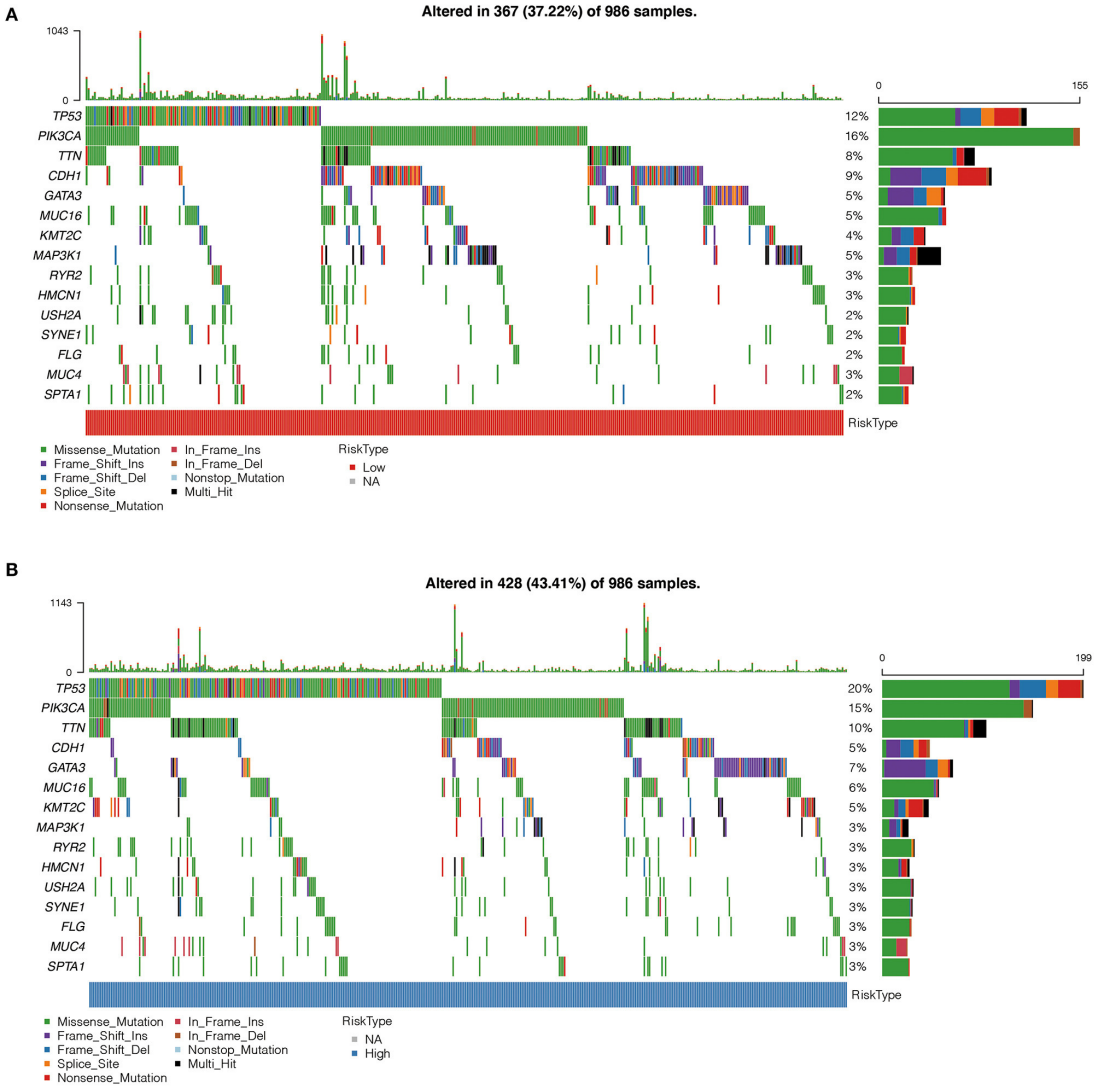
**(A)** Comparison of mutation characteristics in high-risk group. **(B)** Comparison of mutation characteristics in low-risk groups. The abscissa represents the number of samples, and the ordinate represents the mutant gene. Different colors represent different types of mutations.

Using oncoplot to analyze the mutation distributions in the different risk groups, we found that TP53 mutations were significantly more common in the high-risk group than in the low-risk group (20 vs. 12%), and CDH1 mutations were significantly more common in the low-risk group than in the high-risk group (9 vs. 5%). Other mutations, such as PIK3A, TTN, and GATA3 mutations, also had significant differences (Figures 7A,B).

### Nomogram and Decision Curve Analysis of Risk Scores and Clinical Features

**Figure 8:**
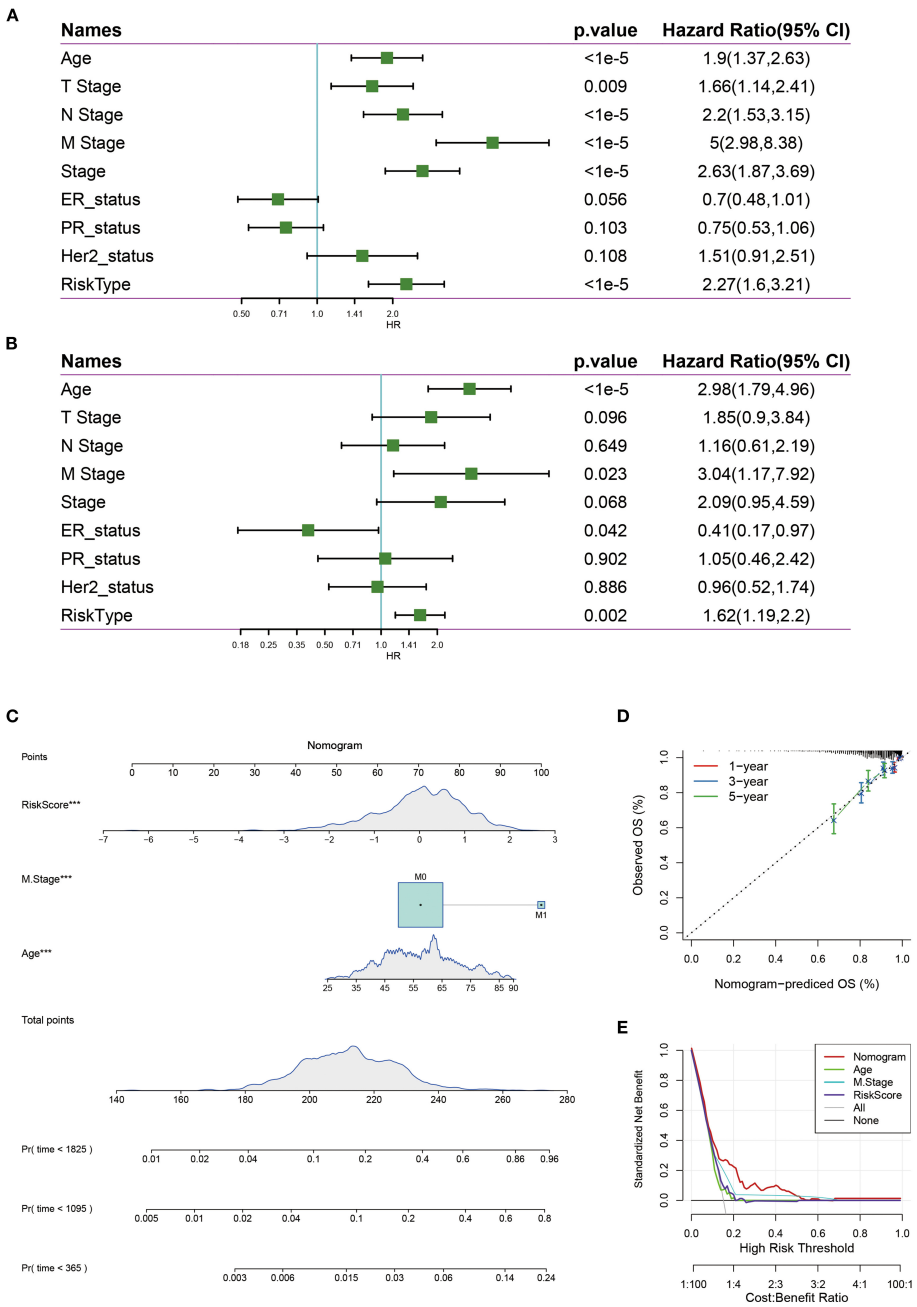
**(A)** Univariate survival analysis of risk score and clinical features; **(B)** Multivariate survival analysis of risk score and clinical features. **(C)** The nomogram model constructed by combining the clinical features with the RiskScore; **(D)** The 1-, 3-, 5- year survival nomogram calibration curves; **(E)** The DCA curves of risk model with clinical features (Age, M Stage, Risk score, and nomogram).

The univariate Cox regression analysis showed that the risk score was significantly associated with the survival of breast cancer patients, while risk type (hazards ratio [HR] = 1.62, *P* = 0.002), age (HR = 2.98, *P* = 1e-05), M stage (HR = 3.04, *P* = 0.023) were significantly related to survival by multivariate Cox regression analysis (Figures 8A,B), suggesting that they were independent risk factors for the prognosis of breast cancer patients.

Nomograms can be used to show risk model results intuitively and effectively, and they are convenient to use in the prediction of outcomes. A nomogram was constructed with the significant factors identified in the multivariate analysis of the entire TCGA data set (Figure 8C). It was can be found that the risk score had the maximum effects on survival and the calibration curves indicated that the risk model had kind predictive performance (Figure 8D). In addition, we plotted the decision curves of age, M Stage, risk score, and the nomogram, and it was found that the nomogram had good clinical applicability (Figure 8E).

### Comparison of the Risk Signature With Others

**Figure 9:**
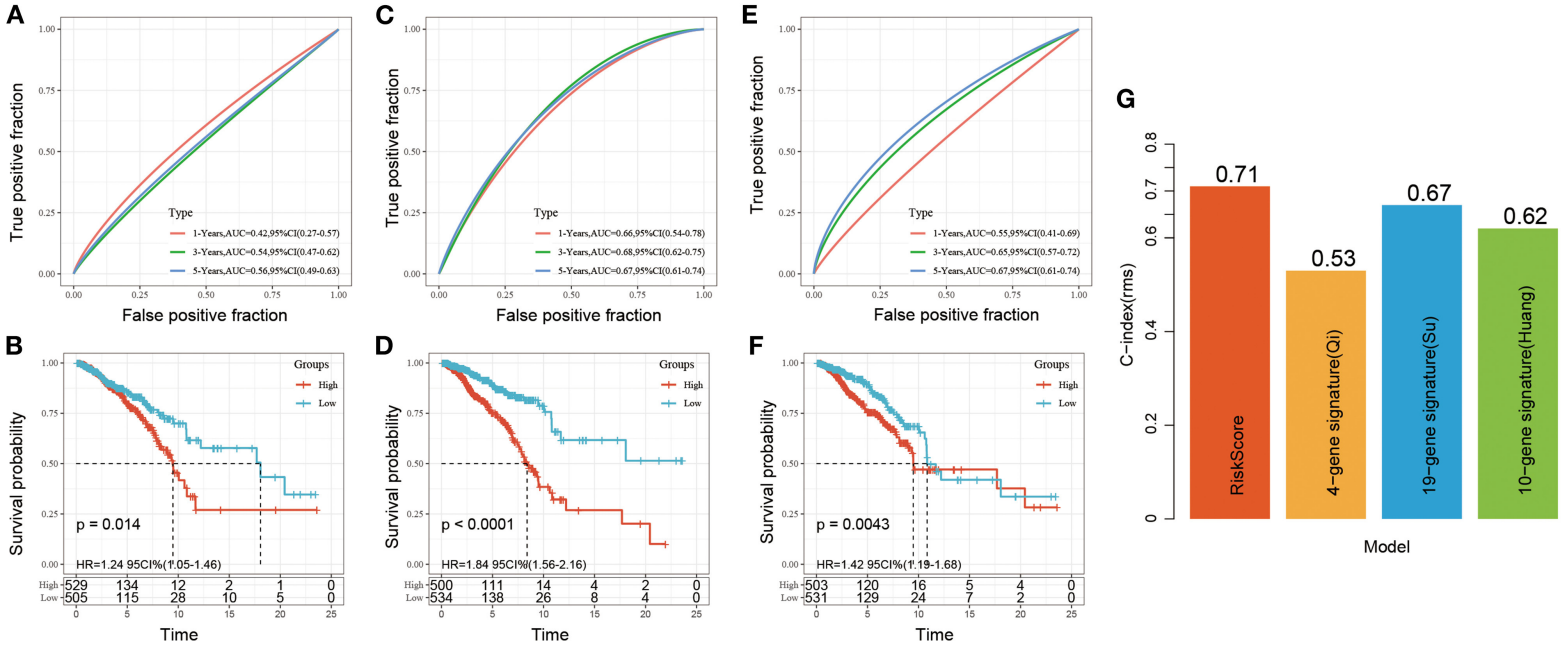
**(A,B)** ROC and KM curve for overall survival for high-risk and low-risk groups based on 4-gene signature (Qi); **(C,D)** ROC and KM curve for overall survival for high-risk and low-risk groups based on 19-gene signature (Su); **(E,F)** ROC and KM curve for overall survival for high-risk and low-risk groups based on 10-gene signature (Huang); **(G)** C-index of four prognostic risk models. The abscissa represents different signatures, and the ordinate represents C-index.

Three risk models about breast cancer prognosis including a 4-gene signature (8), a 19-gene signature (9), and a 10-gene signature (10) were found from the literature. We used the same method to calculate the risk score of each sample according to the corresponding gene in the three models. The ROC and KM curves of the 3 models were shown in Figures 9A–F. The results showed that the 3-year AUC of the 19-gene signature (Su) was higher than our model, but the 1- and 5-year AUCs were lower than those of our model. The 1-, 3-, and 5-year AUCs of the 4-gene signature (Qi) and 10-gene signature (Huang) were lower than those of our 5-gene signature. The KM survival of the high- and low-risk groups was also significantly different between the 3 models (*P* < 0.05). To further compare the predictive performance of the models, we calculated the concordance index (C-index) values of all 4 models with the rms package in R. We found that our risk score model had the highest C-index among the 4 models (Figure 9G), suggesting that the overall performance of our model was better than that of the other 3 models.

### Prediction of Risk Model for Immunotherapy

**Figure 10:**
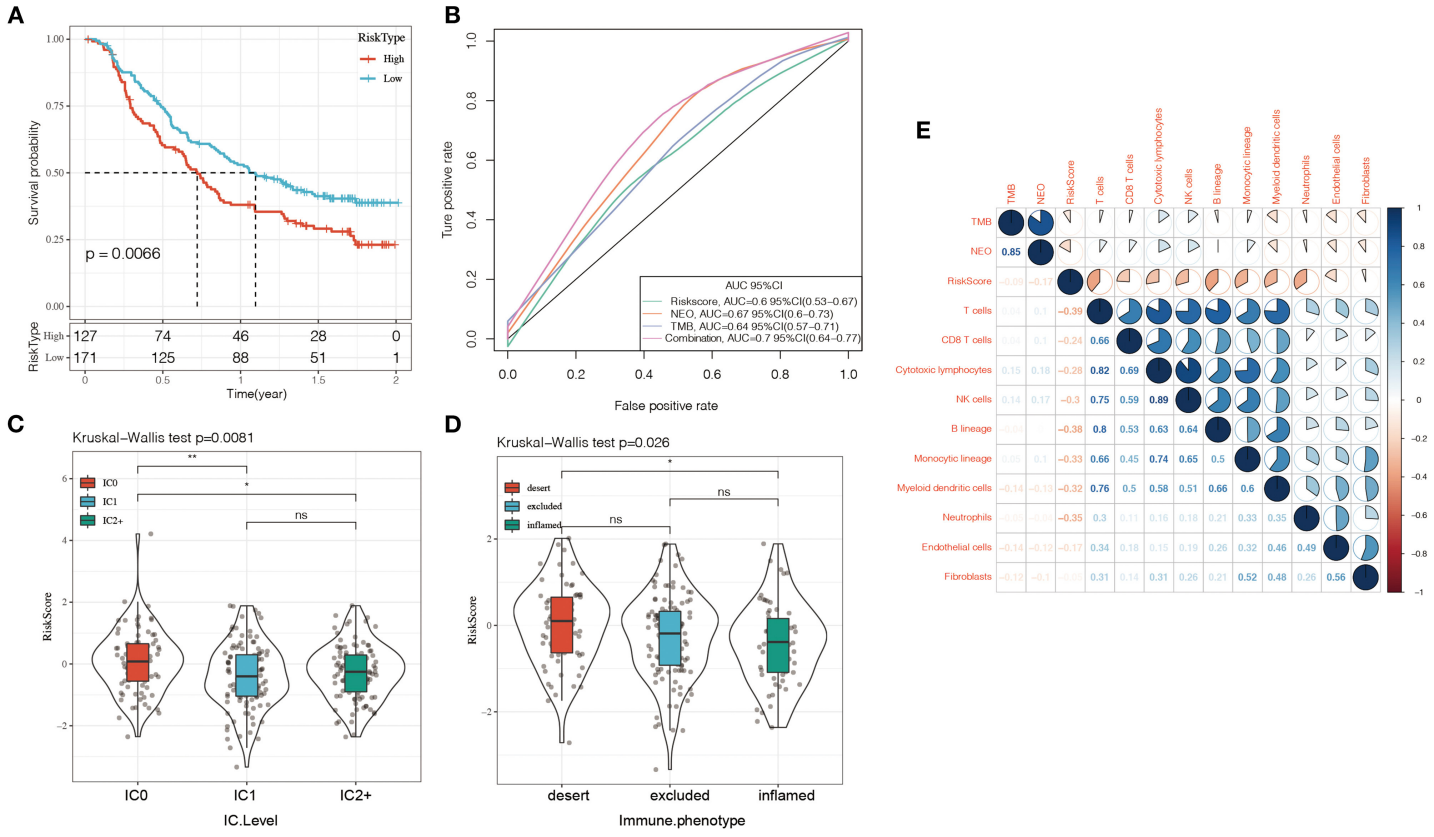
**(A)** KM curve of Imvigor210 data set, horizontal axis represents survival time, vertical axis represents survival probability, red line represents high expression group, green line represents low expression group; **(B)** ROC curve of Imvigor210 data set, horizontal axis represents False postive rate, The vertical axis represents True postive rate; **(C)** RiskScore difference between immune cell (IC) scores, a PD-L1 scoring: IC0: <1%; IC1: ≥ 1% and <5%; IC2: ≥ 5% and <10%; **(D)** Differences in Risk Score between different immune characteristics, The abscissa from left to right is “immune desert” group, “immune excluded” group, and the “inflamed” group; **(E)** Heat map of the correlation between Riskscore and immune characteristic cell type. Red represents positive correlation, blue represents negative correlation.

Currently, effective predictive markers for gene immunotherapy are limited. The identification of new predictive markers is essential for further advancement of precision immunotherapy. We searched an immunotherapy data set (Imvigor210) to explore whether the 5-genes model can predict the effect of immunotherapy. Imvigor210 recorded expression profile in metastatic urothelial carcinoma (mUC) samples from patients who responded or did not respond to anti-PD-L1 immunotherapy. The Kaplan–Meier curve shows that in mUC patients receiving immunotherapy, a higher RiskScore value is associated with a worse survival rate (Figure 10A). ROC analysis shows that the combination model integrating Riskscore, Neo-antigen (NEO), and tumor mutation burden (TMB) has higher predictive performance (Figure 10B, ROC = 0.7); As the immune cell (IC) score increases, the RiskScore gradually decreases (Figure 10C); Tumors were divided into three subgroups, namely “immune desert,” “immune excluded,” and “inflamed” cases in order to recapitulate a proposed model of different immunological backgrounds in cancer. We found that immune-inflamed tumor is often accompanied by low Riskscore (Figure 10D). The expression of Riskscore is negatively correlated with immune cells such as CD8+ Tcell, CD4+ Tcell, B cell (Figure 10E), which further showed that low Riskscore is accompanied by higher immune infiltration. The comprehensive illustration of the above results suggested that the immune response treatment effect of the patients in the low RiskScore group is better than that in the high RiskScore group. Therefore, our model can predict the effect of gene immunotherapy.

### Clinical Validation of 5 Gene Expression

**Figure 11:**
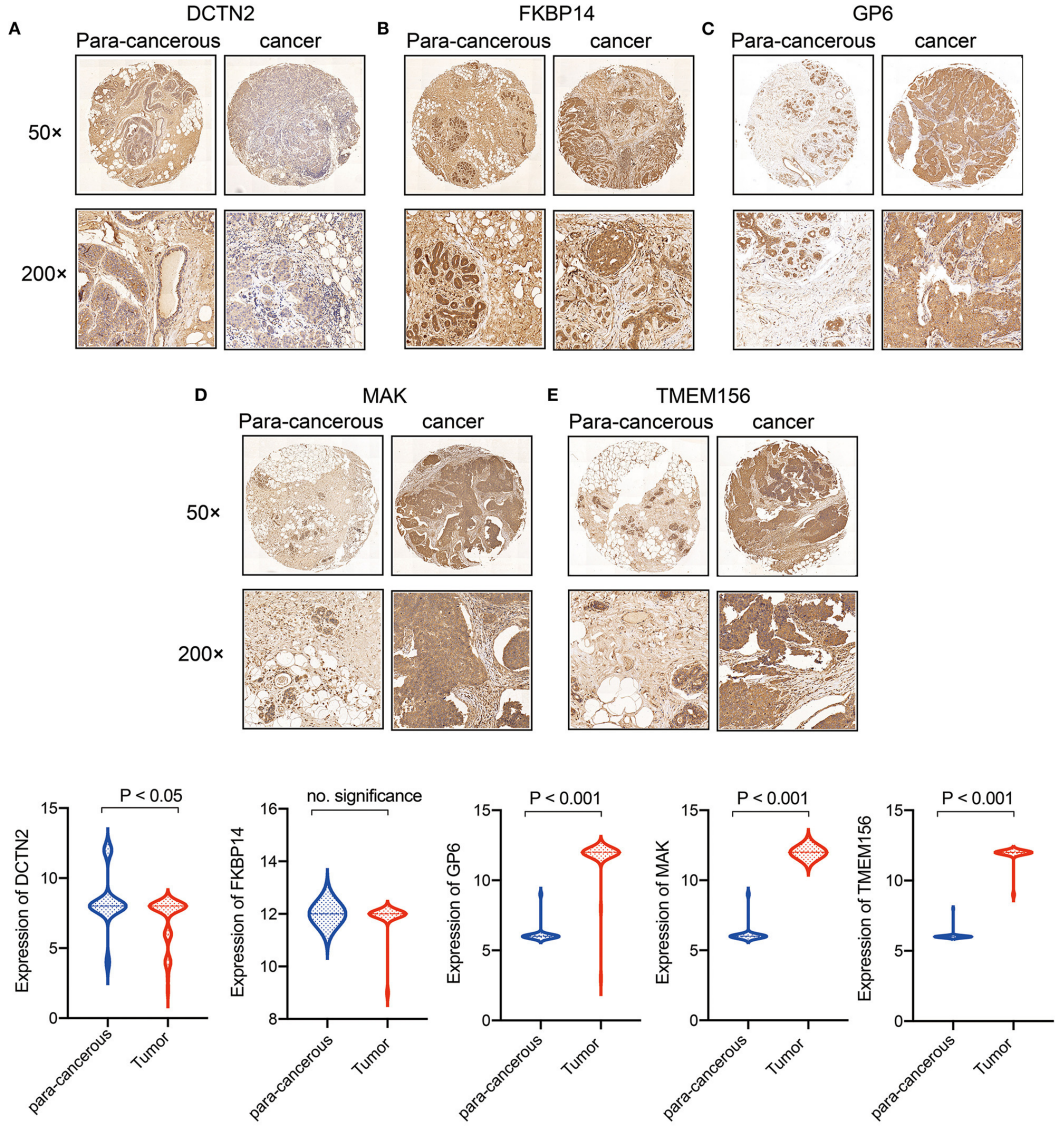
Expression verification of 5 genes in breast cancer clinical samples. **(A)** *DCTN2* expression in cancer and para-cancerous tissues; **(B)** *FKBP14* expression in cancer and para-cancerous tissues; **(C)** *GP6* expression in cancer and para-cancerous tissues; **(D)** *MAK* expression in cancer and para-cancerous tissues; **(E)** *TMEM156* expression in cancer and para-cancerous tissues.

To demonstrate the expression of 5 gene signature further. Tissue microarrays comprised of 89 cases (45 cases of BRCA tissues, 44 cases of normal paired samples) were included. The results of immunohistochemistry showed that MAK, GP6 and TEMEM156 were significantly highly expressed in tumor tissues, and DCTN2 was highly expressed in normal tissues. FKBP14 showed no significant difference between tumor tissues and normal tissues (Figures 11A–E).

## Discussion

Recently, the diagnosis and treatment of breast cancer have improved rapidly due to ongoing research, but assessing and improving prognosis in breast cancer patients are still difficult tasks. Therefore, there is an urgent need to find gene expression biomarkers to help predict prognosis and optimize treatment. To identify such prognostic markers in breast cancer patients, a 5-gene signature was established and validated to investigate the potential link between the risk score and survival.

In the present study, we obtained 1,034 samples of breast cancer from the TCGA database and 180 common DEGs from the intersections of the responder and non-responder subtypes from the GSE59515 and WGCNA. Then AIC was used for stepwise regression to construct a 5-gene signature (GP6, MAK, DCTN2, TMEM156, and FKBP14) as a risk model for survival prediction. The result indicated the 5-gene signature was robust and valid in different data sets (GSE20685 and GSE31448). Compared with the 3 existing models, our 5-gene signature prognostic risk model had better stability and reliability. The analysis revealed that the 5-year survival rate in the high-risk group survived lower than those in the low-risk group. The risk score could divide T stage, N stage, M stage, Clinical stage, ER status, PR status, HER2 status, and the LumB subtype, but not the LumA subtype, into 2 prognostic groups with significance, demonstrating that the predictive power of this model was better than other clinicopathological features included. The risk score was also an independent predictor compared with other clinicopathological features per the 5-year survival nomogram.

Male germ cell-associated kinase (MAK) is the first protein kinase to be shown to be a direct transcriptional target of androgen receptors and to act as a co-activator of androgen receptors in the transmission of androgen signals. MAK is overexpressed in prostate cancer cell lines and clinical specimens and leads to mitosis defects via APC/CCDH1 imbalance. MAK plays important role in normal prostate development and prostate cancer progression. The positive rate of androgen receptors in breast cancer is about 60–80%, and androgen receptors are also key factors in the pathogenesis of breast cancer. Androgen receptor-targeted therapies, including androgen receptor agonists, androgen receptor antagonists, and PI3K inhibitors, have shown encouraging outcomes in breast cancer clinical trials (11). Higher levels of androgen receptor mRNA have been associated with improved survival in patients with ER-positive and HER2-negative breast cancer (12). By inhibiting the expression of MAK, androgen receptor signal transduction is greatly hindered, and the highly restricted expression of this kinase makes it a potential target. Dynactin (DCTN) is a 6-subunit protein encoded by the DCTN gene, which is involved in the activation of cytokinin in eukaryotes (13). This gene is located on chromosome 12q13-q15, which is a region prone to stable amplification in many cancers. The DCTN family has been linked to neurodegeneration (14, 15), and more and more studies have shown that DCTN2 may be a potential prognostic biomarker for cancer, including cutaneous melanoma, colon adenocarcinoma, and osteosarcoma (16–18). FK506-binding protein 14 (FKBP14) belongs to the FK506-binding protein family. FKBP14 is an oncogene that has been reported in several malignant tumors, including osteosarcoma, ovarian cancer, cervical cancer, gastric cancer, and colon cancer. Specifically, FKBP14 acts as an oncogene by inhibiting apoptosis and promoting the movement of human cervical cancer (19). FKBP14 promotes the proliferation and migration of colon cancer cells by targeting the IL-6/STAT3 signaling pathway (20). The expression of FKBP14 is higher in gastric cancer patients with lower survival rates, and it is associated with lymph node metastasis and advanced histological grade. However, reports on FKBP14 in breast cancer are unavailable. Glycoprotein 6 (GP6) is a member of the immunoglobulin superfamily and is believed to be the major platelet collagen receptor involved in arterial thrombosis, and it plays a vital role in platelet activation and aggregation. GP6 has been reported to be associated with the thrombus pathway in acute myocardial infarction, ischemic stroke, and fetal loss (21–23). GP6 and TMEM156 have not been studied in tumors. Further functional studies are needed to explore the molecular functions of these hub genes, and they need to be validated in breast cancer tissue samples or patients.

Breast tumor consisted of tumor cells and stromal cells, such as fibroblasts, endothelial cells, and infiltrating immune cells. The most widely studied immune cell is the tumor infiltrating lymphocyte. The presence of tumor infiltrating lymphocytes is potentially predictive and prognostic in both HER2-positive and triple-negative breast cancer subtypes. Increased tumor infiltrating lymphocyte levels at the time of diagnosis was significantly associated with reduced distant recurrence and good prognosis (24–26). CD8+ tumor infiltrating lymphocytes are particularly essential for tumor destruction. A study of 3,992 breast cancer patients showed that CD8+ tumor infiltrating lymphocytes was an independent indicator related with good survival in patients with basal-like breast cancer, but not patients with other intrinsic molecular subtypes (27). A study of 12,439 patients indicated that the presence of CD8+ T cells dramatically reduced the risk of death in breast cancer (28). Several studies have proven that CD8+ T cells can be used to predict the response to treatment during the neoadjuvant chemotherapy phase, particularly in the triple-negative and HER2-positive breast cancer subtypes (29–31).

Tumor-associated macrophages are related to tumor cell invasion and tumor angiogenesis in breast cancer (32, 33). Tumor-associated macrophages are divided into 2 main phenotypes: M1 macrophages (which inhibit cancer progression) and M2 macrophages (which promote cancer progression). Studies have shown that high concentrations of M2 tumor-associated macrophages and hyaluronic acid in combination lead to inflammatory conditions that promote tumor progression and poor survival (34). CHI3L1 secreted from M2 macrophage promotes breast cancer cell metastasis *in vitro* and *in vivo*. Activation of IL-13Rα2 by CHI3L1 triggers the activation of the mitogen-activated protein kinase signaling pathway, upregulating matrix metalloproteinase genes expression and promoting tumor metasta (35). Besides, M2 macrophages stimulate tumor angiogenesis, cancer cell invasion, immunosuppression, and matrix remodeling (36, 37).

In this study, we calculated immune cell scores using 3 methods and then compared the differences between the high- and low-risk groups. We found that the scores of M0 macrophages and M2 macrophages in the high-risk group were significantly higher than those in the low-risk group. Meanwhile, the scores of B cells and CD8+ T cells in the high-risk group were significantly lower than those in the low-risk group. Per MCPcounter, the scores of B cells and CD8+ T cells in the high-risk group were significantly lower than those in the low-risk group. In coincidence with previous studies: the high expression of M2 macrophages in the high-risk group was linked to bad prognosis in breast cancer, and high expression of CD8+ T cells in the low-risk group was linked to a good prognosis.

Using oncoplot to analyze mutation distributions, we found that TP53 mutations were significantly more common in the high-risk group than in the low-risk group (20 vs. 12%), while CDH1 mutations were significantly more common in the low-risk group than in the high-risk group (9 vs. 5%). TP53 mutations are the most common mutations in breast cancer, occurring in 30–35% of all breast cancer cases and about 80% of triple-negative breast cancer cases (38, 39). There was strong evidence that TP53 mutations were correlated with poor disease-free survival and overall survival rates in breast cancer (40, 41). On the other hand, carriers of mutations in the gene encoding E-cadherin (CDH1) have a significant risk (more than 70%) of developing hereditary diffuse gastric cancer, and women with CDH1 mutations are at high risk (cumulative risk of about 40%) of lobular breast cancer (42). Patients with hereditary diffuse gastric cancer and lobular breast cancer with CDH1 mutations have a poorer prognosis (43, 44). Our findings are consistent with the fact that TP53 mutations are significantly more common in high-risk patients than in low-risk patients, indicating a poor prognosis for breast cancer in those with high-risk scores. However, we found that CDH1 mutations were significantly less common in the high-risk group than in the low-risk group, which was inconsistent with others' findings, possibly due to the limited sample size in this study.

In recent years, there has been more and more research on breast cancer prognosis models. We compared 3 published breast cancer gene signatures to demonstrate the good performance of our model. Huang et al. (10) built and validated a 10-gene signature for breast cancer patients who took tamoxifen. Patients with low-risk scores had significantly longer survival times than those with high risk scores, and the 5-year AUC was 0.733. The risk score was related with stage and grade of lymph node metastasis, but not with age, sex, lymphatic invasion, or tumor size. Qi et al. (8) constructed a 4-mRNA (ACSL1, OTUD3, PKD1L2, and WISP1) prognostic risk model by comprehensive survival analysis. The survival status of the high-risk group was worse than that of the low-risk group. In that study, patients with high expression of OTUD3, PKD1L2, and WISP1 mRNA tended to have a better outcome, while patients with high expression of ACSL1 mRNA tended to have a worse outcome. Su et al. (9) established a 19-gene signature related to clinical prognosis for breast cancer patients. It could also be used to stratify early (I and II) and late (III and IV) breast cancer patients. In this study, we constructed a novel signature and nomogram for breast cancer patients, and it showed good application. The results of ROC analysis and overall survival KM curve analysis of the 4 models showed that the AUC of the 19-gene signature (Su) at 3 years was higher than that of our risk model, but the AUCs at 1 and 5 years were lower than those of our risk model. The AUCs of the 4-gene signature (Qi) and 10-gene signature (Huang) at 1, 3, and 5 years were lower than those of our signature. Besides, Our signature had the highest C-index among the 4 models. These results suggested that our 5-gene signature was superior to the other 3 signatures in terms of overall performance. Further, our signature had a plausible number of genes and had superiority in predicting overall survival.

Our findings were based on retrospective studies, and we did not conduct a comprehensive analysis of the correlations between clinicopathological features and risk scores. This study may have contributed to selection bias because of limited samples, and some key genes may have been omitted leading to the limitation of the risk model.

In conclusion, we identified and constructed a 5-gene signature (GP6, MAK, DCTN2, TMEM156, and FKBP14) prognostic model to predict prognosis in breast cancer patients. The results consistently showed that the survival time of patients with high-risk scores was significantly lower than that of patients with low-risk scores. The model had a good performance in both the training and independent validation cohorts, and it was found to be an independent prognostic clinical feature. Therefore, the 5-gene signature is practical and trustworthy to predict the outcomes of breast cancer patients. We recommend using this classifier as a potential biomarker for the prognosis of breast cancer patients.

## Data Availability Statement

The datasets presented in this study can be found in online repositories. The names of the repository/repositories and accession number(s) can be found in the article/Supplementary Material.

## Author Contributions

LG and YJ designed the current study, collected the data, and analyzed and interpreted the data. LG wrote the manuscript. YJ supervised the study. All authors read and approved the final version of the manuscript and agreed to be accountable for all aspects of the research in ensuring that the accuracy or integrity of any part of the work are appropriately investigated and resolved.

## Funding

This research was granted by Provincial Natural Science Foundation Guidance Program, Functionalized drug-loaded nanovesicles used for tumor targeting US and NIRF dual-modalities imaging and therapy (Project number: 20180550172).

## Conflict of Interest

The authors declare that the research was conducted in the absence of any commercial or financial relationships that could be construed as a potential conflict of interest.

## Publisher's Note

All claims expressed in this article are solely those of the authors and do not necessarily represent those of their affiliated organizations, or those of the publisher, the editors and the reviewers. Any product that may be evaluated in this article, or claim that may be made by its manufacturer, is not guaranteed or endorsed by the publisher.

## References

[1] Siegel RL Miller KD Jemal A. Cancer statistics, 2020. CA Cancer J Clin. (2020) 70:7–30. 10.3322/caac.21590 31912902

[2] Ahmad A. Breast cancer statistics: recent trends. Adv Exp Med Biol. (2019) 1152:1–7. 10.1007/978-3-030-20301-6_1 31456176

[3] DeSantis CE Ma J Gaudet MM Newman LA Miller KD Goding Sauer A. Breast cancer statistics, 2019. CA Cancer J Clin. (2019) 69:438–51. 10.3322/caac.21583 31577379

[4] Rossi L Mazzara C Pagani O. Diagnosis and treatment of breast cancer in young women. Curr Treat Options Oncol. (2019) 20:86. 10.1007/s11864-019-0685-7 31776799

[5] Zhong S Chen H Yang S Feng J Zhou S. Identification and validation of prognostic signature for breast cancer based on genes potentially involved in autophagy. PeerJ. (2020) 8:e9621. 10.7717/peerj.9621 33194339 PMC7391974

[6] Zhang D Zheng Y Yang S Li Y Wang M Yao J. Identification of a novel glycolysis-related gene signature for predicting breast cancer survival. Front Oncol. (2020) 10:596087. 10.3389/fonc.2020.596087 33489894 PMC7821871

[7] Xie P Ma Y Yu S An R He J Zhang H. Development of an immune-related prognostic signature in breast cancer. Front Genet. (2019) 10:1390. 10.3389/fgene.2019.01390 32047513 PMC6997532

[8] Qi L Yao Y Zhang T Feng F Zhou C Xu X. A four-mRNA model to improve the prediction of breast cancer prognosis. Gene. (2019) 721:144100. 10.1016/j.gene.2019.144100 31493508

[9] Su J Miao LF Ye XH Cui MS He XF. Development of prognostic signature and nomogram for patients with breast cancer. Medicine. (2019) 98:e14617. 10.1097/MD.0000000000014617 30882627 PMC6426514

[10] Huang H Chen Q Sun W Lu M Yu Y Zheng Z. Expression signature of ten genes predicts the survival of patients with estrogen receptor positive-breast cancer that were treated with tamoxifen. Oncol Lett. (2018) 16:573–9. 10.3892/ol.2018.8663 29928444 PMC6006464

[11] Kono M Fujii T Lim B Karuturi MS Tripathy D Ueno NT. Androgen receptor function and androgen receptor-targeted therapies in breast cancer: a review. JAMA Oncol. (2017) 3:1266–73. 10.1001/jamaoncol.2016.4975 28301631

[12] Venema CM Bense RD Steenbruggen TG Nienhuis HH Qiu SQ van Kruchten M. Consideration of breast cancer subtype in targeting the androgen receptor. Pharmacol Ther. (2019) 200:135–47. 10.1016/j.pharmthera.2019.05.005 31077689

[13] Cianfrocco MA DeSantis ME Leschziner AE Reck-Peterson SL. Mechanism and regulation of cytoplasmic dynein. Annu Rev Cell Dev Biol. (2015) 31:83–108. 10.1146/annurev-cellbio-100814-125438 26436706 PMC4644480

[14] Heiman-Patterson TD Blankenhorn EP Sher RB Jiang J Welsh P Dixon MC. Genetic background effects on disease onset and lifespan of the mutant dynactin p150Glued mouse model of motor neuron disease. PLoS ONE. (2015) 10:e0117848. 10.1371/journal.pone.0117848 25763819 PMC4357475

[15] Steele JC Guella I Szu-Tu C Lin MK Thompson C Evans DM. Defining neurodegeneration on Guam by targeted genomic sequencing. Ann Neurol. (2015) 77:458–68. 10.1002/ana.24346 25558820

[16] Bransfield KL Askham JM Leek JP Robinson PA Mighell AJ. Phenotypic changes associated with DYNACTIN-2 (DCTN2) over expression characterise SJSA-1 osteosarcoma cells. Mol Carcinog. (2006) 45:157–63. 10.1002/mc.20151 16369996

[17] Wang Q Wang X Liang Q Wang S Liao X Li D. Prognostic value of dynactin mRNA expression in cutaneous melanoma. Med Sci Monit Int Med J Exp Clin Res. (2018) 24:3752–63. 10.12659/MSM.910566 29864111 PMC6016438

[18] Wang S Wang Q Zhang X Liao X Wang G Yu L. Distinct prognostic value of dynactin subunit 4 (DCTN4) and diagnostic value of DCTN1, DCTN2, and DCTN4 in colon adenocarcinoma. Cancer Manag Res. (2018) 10:5807–24. 10.2147/CMAR.S183062 30510450 PMC6248376

[19] Sun LY Tao JZ Yan B Lin JS. Inhibitory effects of FKBP14 on human cervical cancer cells. Mol Med Rep. (2017) 16:4265–72. 10.3892/mmr.2017.7043 28731139

[20] Yang L Zhang R Yang J Bi T Zhou S. FKBP14 promotes the proliferation and migration of colon carcinoma cells through targeting IL-6/STAT3 signaling pathway. Onco Targets Ther. (2019) 12:9069–76. 10.2147/OTT.S222555 31802914 PMC6830384

[21] Croft SA Samani NJ Teare MD Hampton KK Steeds RP Channer KS. Novel platelet membrane glycoprotein VI dimorphism is a risk factor for myocardial infarction. Circulation. (2001) 104:1459–63. 10.1161/hc3801.096397 11571236

[22] Induruwa I Jung SM Warburton EA. Beyond antiplatelets: the role of glycoprotein VI in ischemic stroke. Int J Stroke. (2016) 11:618–25. 10.1177/1747493016654532 27312676 PMC5390959

[23] Shaffer JR Kammerer CM Dorn J Ferrell RE Iacoviello L Trevisan M. Polymorphisms in the platelet-specific collagen receptor GP6 are associated with risk of nonfatal myocardial infarction in Caucasians. Nutr Metab Cardiovasc Dis. (2011) 21:546–52. 10.1016/j.numecd.2009.12.002 20227257 PMC2888832

[24] Loi S Michiels S Salgado R Sirtaine N Jose V Fumagalli D. Tumor infiltrating lymphocytes are prognostic in triple negative breast cancer and predictive for trastuzumab benefit in early breast cancer: results from the FinHER trial. Ann Oncol. (2014) 25:1544–50. 10.1093/annonc/mdu112 24608200

[25] Loi S Sirtaine N Piette F Salgado R Viale G Van Eenoo F. Prognostic and predictive value of tumor-infiltrating lymphocytes in a phase III randomized adjuvant breast cancer trial in node-positive breast cancer comparing the addition of docetaxel to doxorubicin with doxorubicin-based chemotherapy: BIG 02-98. J Clin Oncol. (2013) 31:860–7. 10.1200/JCO.2011.41.0902 23341518

[26] Salgado R Denkert C Campbell C Savas P Nuciforo P Aura C. Tumor-infiltrating lymphocytes and associations with pathological complete response and event-free survival in HER2-Positive early-stage breast cancer treated with lapatinib and trastuzumab: a secondary analysis of the NeoALTTO trial. JAMA Oncol. (2015) 1:448–54. 10.1001/jamaoncol.2015.0830 26181252 PMC5551492

[27] Liu S Lachapelle J Leung S Gao D Foulkes WD Nielsen TO. CD8+ lymphocyte infiltration is an independent favorable prognostic indicator in basal-like breast cancer. Breast Cancer Res. (2012) 14:R48. 10.1186/bcr3148 22420471 PMC3446382

[28] Ali HR Provenzano E Dawson SJ Blows FM Liu B Shah M. Association between CD8+ T-cell infiltration and breast cancer survival in 12,439 patients. Ann Oncol. (2014) 25:1536–43. 10.1093/annonc/mdu191 24915873

[29] Lee HJ Seo JY Ahn JH Ahn SH Gong G. Tumor-associated lymphocytes predict response to neoadjuvant chemotherapy in breast cancer patients. J Breast Cancer. (2013) 16:32–9. 10.4048/jbc.2013.16.1.32 23593079 PMC3625767

[30] Mahmoud SM Paish EC Powe DG Macmillan RD Grainge MJ Lee AH. Tumor-infiltrating CD8+ lymphocytes predict clinical outcome in breast cancer. J Clin Oncol. (2011) 29:1949–55. 10.1200/JCO.2010.30.5037 21483002

[31] Vihervuori H Autere TA Repo H Kurki S Kallio L Lintunen MM. Tumor-infiltrating lymphocytes and CD8(+) T cells predict survival of triple-negative breast cancer. J Cancer Res Clin Oncol. (2019) 145:3105–14. 10.1007/s00432-019-03036-5 31562550 PMC6861359

[32] Choi J Gyamfi J Jang H Koo JS. The role of tumor-associated macrophage in breast cancer biology. Histol Histopathol. (2018) 33:133–45. 10.14670/HH-11-916 28681373

[33] Qiu SQ Waaijer SJH Zwager MC de Vries EGE van der Vegt B Schröder CP. Tumor-associated macrophages in breast cancer: innocent bystander or important player? Cancer Treat Rev. (2018) 70:178–89. 10.1016/j.ctrv.2018.08.010 30227299

[34] Tiainen S Masarwah A Oikari S Rilla K Hämäläinen K Sudah M. Tumor microenvironment and breast cancer survival: combined effects of breast fat, M2 macrophages and hyaluronan create a dismal prognosis. Breast Cancer Res Treat. (2020) 179:565–75. 10.1007/s10549-019-05491-7 31720917 PMC6997252

[35] Chen Y Zhang S Wang Q Zhang X. Tumor-recruited M2 macrophages promote gastric and breast cancer metastasis via M2 macrophage-secreted CHI3L1 protein. J Hematol Oncol. (2017) 10:36. 10.1186/s13045-017-0408-0 28143526 PMC5286803

[36] Goswami KK Ghosh T Ghosh S Sarkar M Bose A Baral R. Tumor promoting role of anti-tumor macrophages in tumor microenvironment. Cell Immunol. (2017) 316:1–10. 10.1016/j.cellimm.2017.04.005 28433198

[37] Santoni M Romagnoli E Saladino T Foghini L Guarino S Capponi M. Triple negative breast cancer: key role of tumor-associated macrophages in regulating the activity of anti-PD-1/PD-L1 agents. Biochim Biophys Rev Cancer. (2018) 1869:78–84. 10.1016/j.bbcan.2017.10.007 29126881

[38] Duffy MJ Synnott NC Crown J. Mutant p53 in breast cancer: potential as a therapeutic target and biomarker. Breast Cancer Res Treat. (2018) 170:213–9. 10.1007/s10549-018-4753-7 29564741

[39] Silwal-Pandit L Vollan HK Chin SF Rueda OM McKinney S Osako T. TP53 mutation spectrum in breast cancer is subtype specific and has distinct prognostic relevance. Clin Cancer Res. (2014) 20:3569–80. 10.1158/1078-0432.CCR-13-2943 24803582

[40] Olivier M Langerød A Carrieri P Bergh J Klaar S Eyfjord J. The clinical value of somatic TP53 gene mutations in 1,794 patients with breast cancer. Clin Cancer Res. (2006) 12:1157–67. 10.1158/1078-0432.CCR-05-1029 16489069

[41] Silwal-Pandit L Langerød A Børresen-Dale AL. TP53 mutations in breast and ovarian cancer. Cold Spring Harb Perspect Med. (2017) 7:a026252. 10.1101/cshperspect.a026252 27815305 PMC5204332

[42] Pharoah PD Guilford P Caldas C. Incidence of gastric cancer and breast cancer in CDH1 (E-cadherin) mutation carriers from hereditary diffuse gastric cancer families. Gastroenterology. (2001) 121:1348–53. 10.1053/gast.2001.29611 11729114

[43] Corso G Intra M Trentin C Veronesi P Galimberti V. CDH1 germline mutations and hereditary lobular breast cancer. Fam Cancer. (2016) 15:215–9. 10.1007/s10689-016-9869-5 26759166

[44] Hansford S Kaurah P Li-Chang H Woo M Senz J Pinheiro H. Hereditary diffuse gastric cancer syndrome: CDH1 mutations and beyond. JAMA Oncol. (2015) 1:23–32. 10.1001/jamaoncol.2014.168 26182300

