# Supplementary figures and images for: Construction and Identification of a Novel 5-Gene Signature for Predicting the Prognosis in Breast Cancer
Source: Front Med (Lausanne). 2021 Oct 14;8:669931. doi: 10.3389/fmed.2021.669931 (PMC8551811; doi:10.3389/fmed.2021.669931)

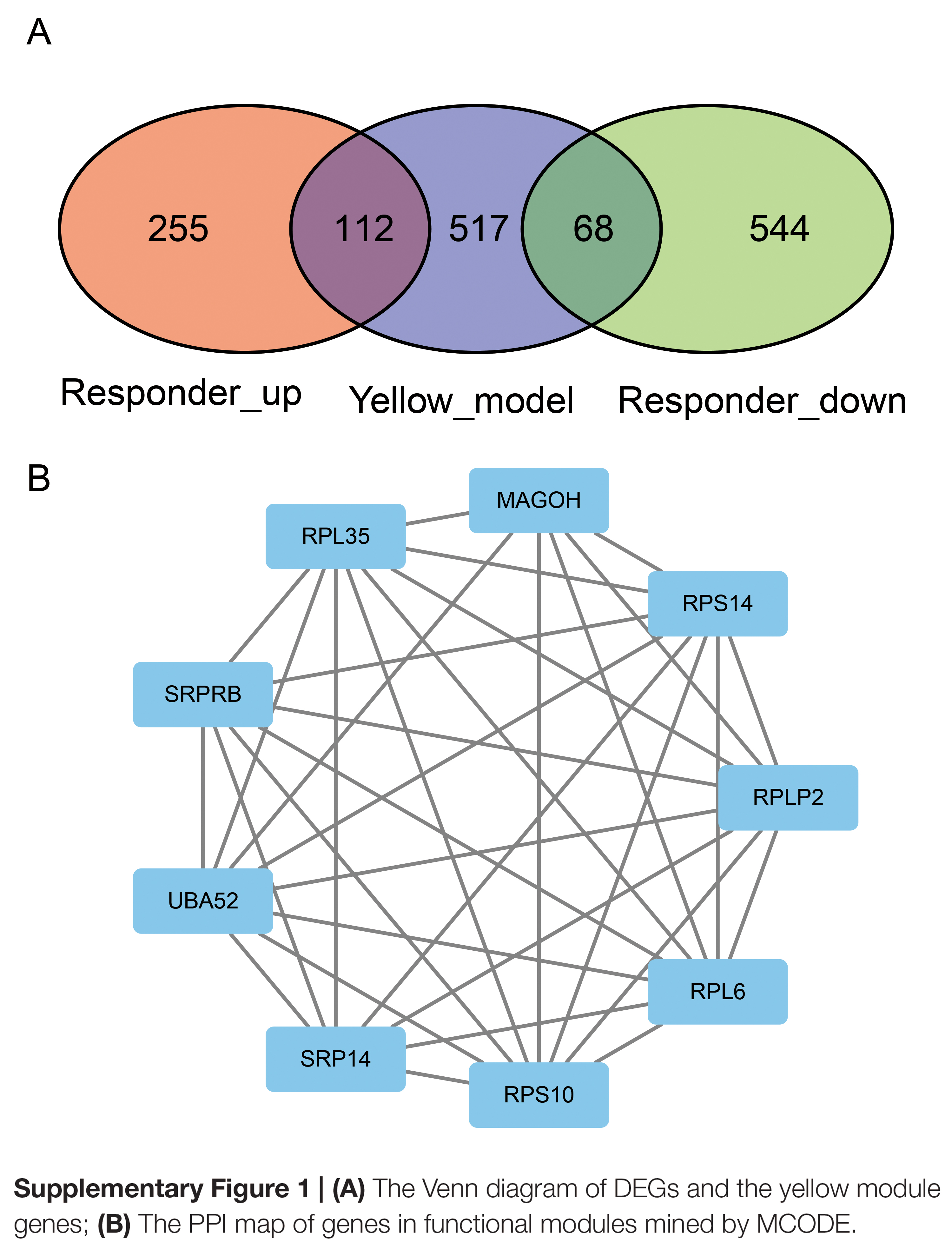

Supplement: Supplementary file 7 [file Image_1.JPEG]

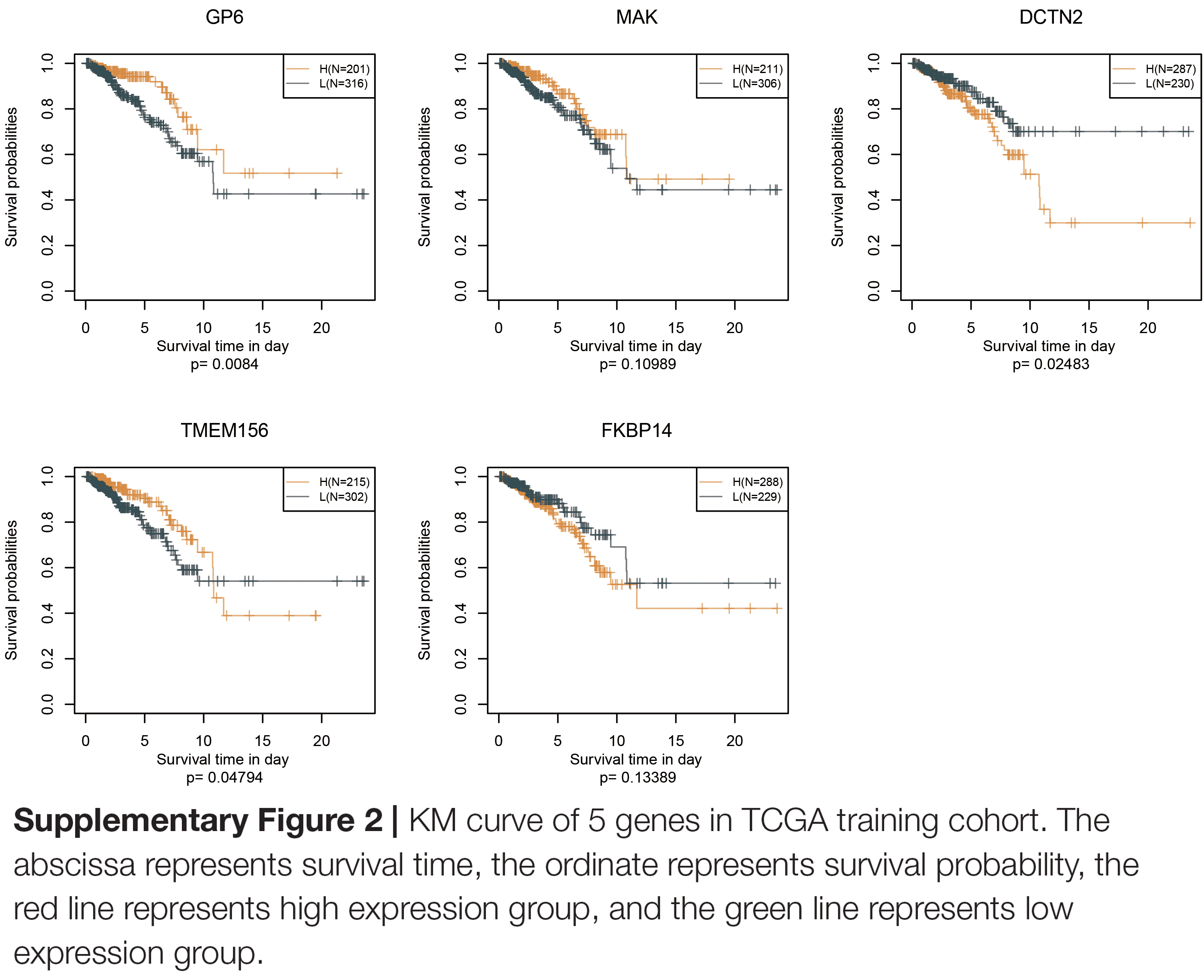

Supplement: Supplementary file 8 [file Image_2.JPEG]

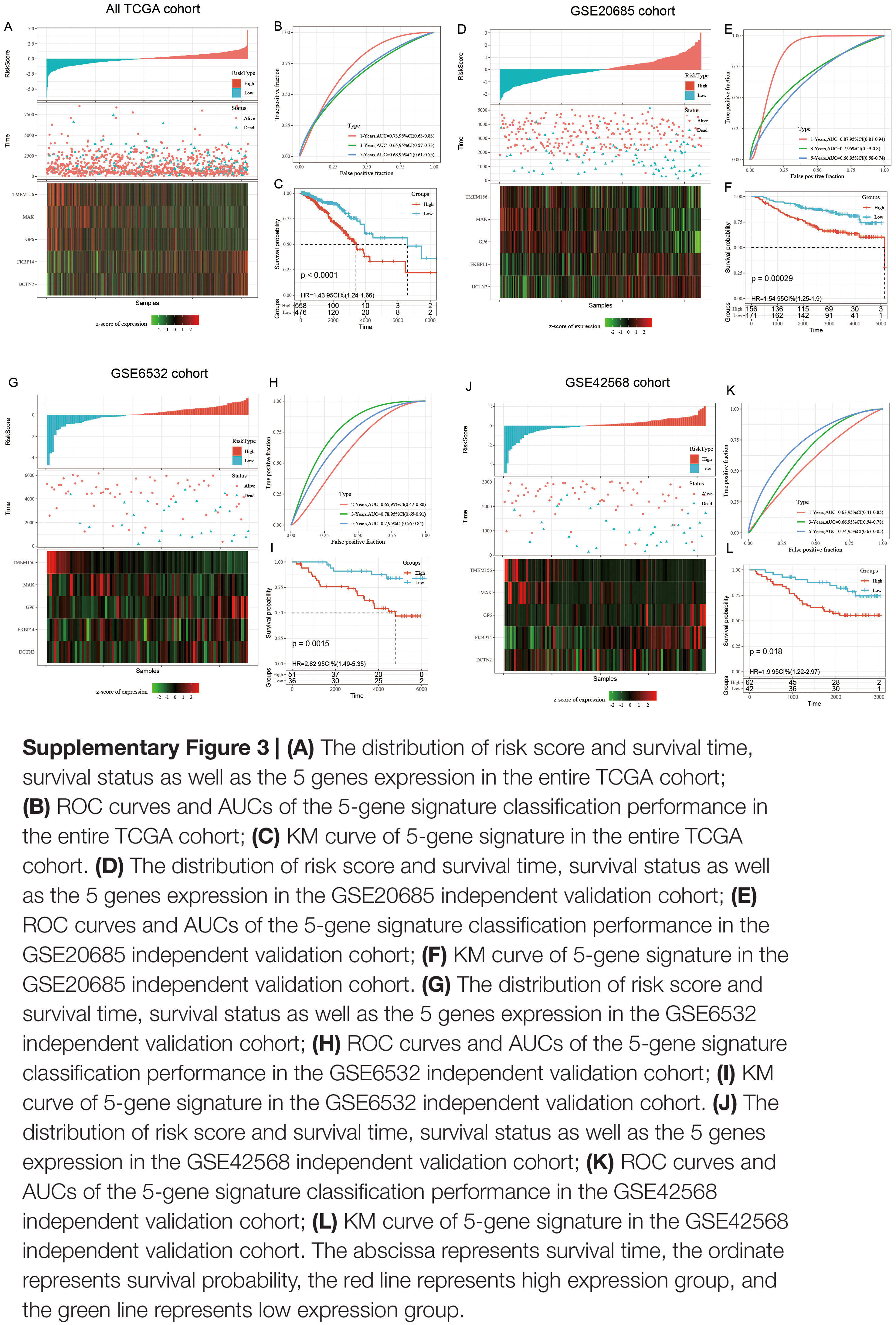

Supplement: Supplementary file 9 [file Image_3.JPEG]
